# Mechanical Property of Thermoplastic Polyurethane Vascular Stents Fabricated by Fused Filament Fabrication

**DOI:** 10.3390/mi15101266

**Published:** 2024-10-17

**Authors:** Yun Zhai, Zezhi Sun, Tie Zhang, Changchun Zhou, Xiangpeng Kong

**Affiliations:** 1 School of Mechanical Engineering, Dalian Jiaotong University, Dalian 116028, China; yzhai@djtu.edu.cn (Y.Z.);; 2 National Engineering Research Centre for Biomaterials, College of Biomedical Engineering, Sichuan University, Chengdu 610065, China; changchunzhou@scu.edu.cn; 3 Department of Cardiology, The Second Hospital of Dalian Medical University, Dalian 116023, China

**Keywords:** fused filament fabrication, vascular stent, mechanical property, unit structure

## Abstract

Vascular stents have many applications in treating arterial stenosis and other vascular-related diseases. The ideal vascular stent for clinical application should have radial support and axial bending mechanical properties that meet the requirements of vascular deformation coordination. The materials used for vascular stents implanted in the human body should have corresponding biocompatibility to ensure that the stents do not cause coagulation, hemolysis, and other reactions in the blood. This study fabricated four types of vascular stents, including inner hexagon, arrowhead, quadrilateral, and outer hexagonal, using fused filament fabrication technology and thermoplastic polyurethane (TPU) as materials. By evaluating the effects of edge width and wall thickness on the radial support and axial bending performance, it was found that the inner hexagonal stent exhibited the best radial support and axial bending performance under the same conditions. The design and fabrication of vascular stents based on 3D printing technology have promising application prospects in personalized customized vascular repair therapy.

## 1. Introduction

Aortic aneurysms [1], iliac artery stenosis and occlusion [2], and subclavian artery stenosis [3] are common vascular diseases. The clinical treatment of these diseases often involves using vascular stents to expand the blood vessels and ensure normal blood flow effectively. The diameters of the blood vessels mentioned above range from 7.3 to 23.98 mm, and the outer diameter of the implanted vascular stents can be slightly larger than the vessel diameter to ensure effective expansion and support [4,5]. This paper proposes tubes warped from 2D unit structure arrays based on TPU material. Compared to the covered metallic stents [6], TPU material offers better flexibility and elasticity, allowing it to accommodate blood vessels’ natural movement and deformation. In addition, TPU material has excellent biocompatibility, which can reduce the body’s rejection and inflammatory responses [7]. 3D printing technology for TPU materials can provide personalized geometric customization and rapid prototyping for complexly structured diseased blood vessels.

Poly(acid lactic) (PLA) and its mixtures are commonly used for vascular stents in the medical field [8]. The integration of PLA and bio fibers into biocomposites through transversely grown ZnO nanowires provides a significant increase in interfacial shear strength and debonding energy compared to virgin materials, guiding large-scale applications [9]. When printing with PLA materials, there is a synergy between different print processing parameters, such as melt temperature, processing speed, and layer height, to create a homogeneous, high-finish, and durable polymer structure [10]. Wu et al. enhanced PLA stent performance through 3D printing, improving radial support and demonstrating shape memory effects [11]. Tong et al. further improved the physical properties of PLA stents with unique heat treatment [12]. Nevertheless, as a brittle material, PLA is prone to fracture, which may lead to stent failure and secondary vascular injury [13]. The traditional processing of vascular stents involves laser cutting, planar lithography, welding, injection molding, etc., each with drawbacks such as rough surfaces, heat-affected zones, fracture risks, and complex mold requirements [14,15,16]. Additive manufacturing, also known as 3D printing, overcomes these issues by a layer-by-layer deposition of materials to produce complex shapes [17,18,19]. It offers personalized design, rapid production, and improved treatment efficiency, with the ability to select suitable biomaterials for individual blood vessels, reducing thrombosis and restenosis risks [20,21,22]. Among many additive manufacturing technologies, a 3D printing method called fused filament fabrication (FFF) has become increasingly popular in the medical field [23]. FFF technology exhibits significant advantages for fabricating vascular stents due to its relatively high single-part processing efficiency and low consumable costs [24]. However, it inevitably has some weaknesses. Probably the most important is the low mechanical properties of the parts obtained by this technique. This weakness can be overcome by resorting to post-treatment procedures, adding different types of reinforcements, and optimizing the printing parameters [25]. Robert van Lith et al. invented a vascular stent structure with high support performance and low flexibility [26]. The balance between support stiffness and bending compliance remains a critical unsolved issue. Stress scenario-oriented deformation coordination is used in engineering to solve bearing problems [27]. The principle requires reducing rigidity at the contact area and enabling synchronous deformation to avoid stress concentration [28]. Upon implantation, the stent interacts with the vessel’s inner wall, deforming correspondingly [29]. Compliance measures the stent’s ability to follow vascular deformation. A high-compliance stent effectively meets stress and deformation coordination conditions, reducing damage to the vessel’s inner wall due to stress concentration [30]. The negative Poisson’s ratio structure (NPR) has unique mechanical properties, enabling novel applications [31,32]. It exhibits lateral contraction under uniaxial pressure and advantages over traditional structures in shear-bearing capacity [33], fracture resistance, energy absorption [34,35], and collapse resistance [36]. Thus, NPR materials have broad application prospects in medical equipment [37]. The mechanical property of vascular stent is a critical factor affecting its therapeutic effect. The success of the implantation operation requires that the vascular stent has good radial support performance to ensure the average flow rate of the blood. At the same time, the blood vessels need to have good bending flexibility to ensure they can deform and coordinate with the blood vessels at the implantation site. The optimization of the balance between the support stiffness and bending compliance for the working condition has become a critical scientific problem that still needs to be solved. The smaller the wall thickness, the better the stent compliance, and the greater the space for blood flow in the tube. However, if the wall thickness is too small, the radial support strength of the stent will be insufficient. Therefore, under the premise of satisfying the support strength, a relatively smaller wall thickness is preferred.

Through 3D printing, it becomes feasible to create structures with complex geometries that prove difficult to achieve through traditional manufacturing techniques. Three-dimensional printing enables medical devices to cater to individual patient requirements. This personalized approach leads to more effective and targeted treatments, improving patient outcomes [38]. The 3D printing of dynamic cross-linked polymers is an innovative technology that combines additive manufacturing technology with the properties of dynamic cross-linked polymers. This technology not only improves the printability of the material but also enables the reprogramming of the material’s macroscopic properties, such as self-healing, processability, and recyclability at the post-synthesis stage [39]. The unique dynamic covalent chemistry design makes it possible to print multiple products and reconfigure geometries simultaneously [40].

The ideal vascular stent should be non-toxic and stable in physicochemical properties. Researchers prefer selecting better materials [41] and drugs [42] to adapt to the human blood vessel environment, reducing post-implantation inflammation. TPU materials exhibit good flexibility and are less likely to cause vascular damage. With excellent blood compatibility and biocompatibility, high elasticity, high tensile strength, low-temperature adaptability, wear resistance, and corrosion resistance, TPU materials show promising application prospects in the field of medical devices [43,44]. Currently, more than half of the related research is devoted to developing medical devices such as pacemakers, heart valves, artificial blood vessels, and breast implants using medical-grade polyurethane [45].

In this study, vascular stents with four types of unit structures, including inner hexagonal, arrow-shaped, quadrilateral, and hexagonal, were designed and manufactured by the fused filament fabrication process using TPU material. The influence of the edge width of the unit structure and the thickness of the stent wall on the overall radial support performance and axial compliance performance of the vascular stent was explored. The finite element simulation and experimental test jointly verified the influence of vascular stent structure on mechanical properties. This paper explores the evolution between the unit structure and macroscopic mechanical properties of TPU materials based on 3D printing, which provides a reference for the personalized repair of diseases such as aortic aneurysms, iliac artery stenosis and occlusion, and subclavian artery stenosis.

## 2. Structural Design and Fabrication

### 2.1. Selection of Vascular Stent Unit Structures

Different vascular stents are composed of different stent units [46]. Different vascular stents with different stent units have different mechanical properties [47,48]. A variation in the design parameters of a stent unit improves the performance of vascular stents [49,50]. However, there are few studies based on the types of stent units to verify the compliance and support performance of different stents [51], which makes us lack adequate references when choosing stent types. As a result, the most suitable stent cannot be obtained. This study selected four types of stent elements for the optimal design, including inner hexagonal, arrow, quadrilateral, and hexagonal elements.

The quadrilateral unit gives excellent stability and strength. The structure with four sides and four vertices can effectively distribute and withstand loads from all directions. The arrow-shaped unit has good structural stability. When subjected to external loads, the stress is dispersed along the various parts of the arrow-shaped structure, reducing the possibility of a single point of force and improving the stability of the overall structure. The hexagonal unit evenly distributes the pressure on all sides, thus providing a stable radial support force that can withstand large external forces without damage. The inner hexagonal unit is a negative Poisson’s ratio structure, which undergoes lateral contraction (expansion) under uniaxial pressure (tensile force), which has advantages over traditional structures in terms of shear resistance, fracture resistance, energy absorption, and depression resistance.

**Figure 1:**
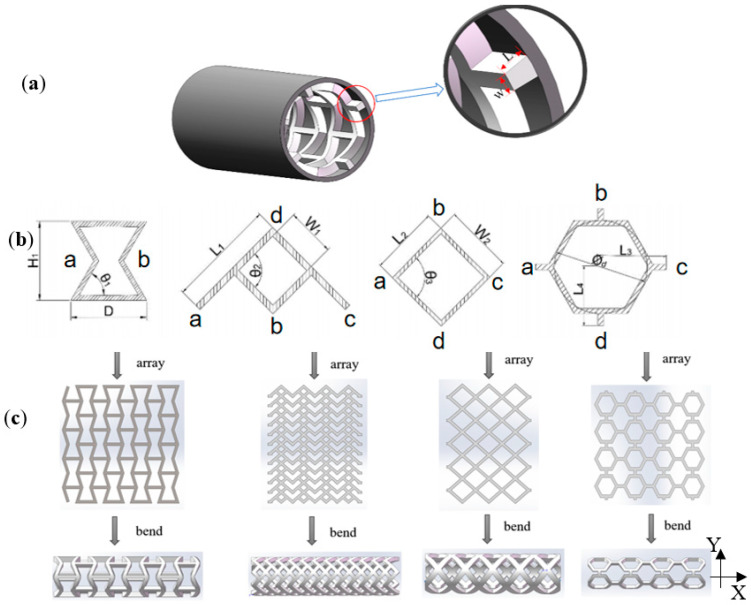
Models of vascular stents in this work. (**a**) The definition of the edge width, W, and the wall thickness, L, of the stent; (**b**) from left to right: hexagon unit inside, arrow unit, quad unit, and hexagon unit; (**c**) the buildup of the 3D modeling of four types of stents.

On the other hand, the design parameters of different stent units are very different. However, the edge width and the wall thickness are the common characteristics of each stent—four types of stents under the condition of fixed design parameters of the stent unit. We took the edge width, W, and the wall thickness, L, as the geometric parameter variables to study their influence on the mechanical properties of four types of vascular stents. The definition of W and L is shown in Figure 1a. After trial and estimation, the macroscopic mechanical properties of vascular stents are roughly consistent with those of human blood vessels when the edge width range is 1.0–1.6 mm and the wall thickness range is 1.0 to 1.3 mm. The three-dimensional model was established by the computer-aided design software Solidworks Premium 2021 SP0.0.

The establishment of the stent model entails that a single stent unit is arrayed to form a planar porous structure. Then, the array object is bent around the horizontal axis into a cylindrical thin-walled vascular stent. The three-dimensional model-building process of the four types of stents is shown in Figure 1c. From left to right, there are inner hexagonal stents, arrow stents, quadrilateral stents, and hexagonal stents.

The inner hexagon structural elements are arrayed along the *Y*-axis and the *X*-axis. Points *a* and *b* are connected by a horizontal beam with the length of *D* to form a two-dimensional structure. The array replicates infinitely along the mutually perpendicular *X*-axis and *Y*-axis to form a planar porous structure, which is annularly bent around the *X*-axis. The three-dimensional model of the inner hexagonal tubular structure is built accordingly.

The array of the arrow-shaped structural element along the *X*-axis direction is connected by points *a* and *c*. The array along the Y-axis direction is connected by points *d* and *b* to form a plane pattern. The array replicates infinitely along the mutually perpendicular *X*-axis and *Y*-axis to form a planar porous structure, which is annularly bent around the *Y*-axis. A three-dimensional model of the arrow-shaped tubular structure is established.

The array of the quadrilateral structural element along the *X*-axis direction is connected by points *a* and *c*. The array along the *Y*-axis direction is connected by points *d* and *b* to form a plane figure. Then, the array replicates infinitely along the mutually perpendicular *X*-axis and *Y*-axis to form a planar porous structure. The planar porous structure is annularly bent around the *Y*-axis to establish a three-dimensional model of the quadrilateral tubular structure.

The array of hexagonal structural elements along the *X*-axis direction is connected by points *a* and *c*. The array along the *Y*-axis direction is connected by points *d* and *b* to form a plane structure. The array replicates infinitely along the mutually perpendicular *X*-axis and *Y*-axis to form a planar porous structure, which is annularly bent around the *X*-axis. A three-dimensional model of the hexagonal tubular structure is established.

Some design parameters of each vascular stent unit are defined as the following: *W_1_* is the length of the short side of the arrow unit, which is equal to four short sides. *W_2_* is the quadrilateral unit’s side length, equal to *L*_2_. *L*_1_ is the length of the long side of the arrow element. *L*_3_ is the length of the center of the inscribed circle of the hexagonal unit to the side of the unit structure. *L*_4_ is the length of the center of the hexagonal unit’s inscribed circle to the unit structure’s bottom edge. The geometric dimension parameters are summarized in Table 1.

The printing accuracy of 3D printing equipment cannot meet the accuracy requirements of vascular stents. The minimum printing size we control is the stent’s outer and inner diameters, which are 12.8 mm and 10.4 mm. The decreasing of the printing size of the sample might lead to a relatively low quality of the stent. Removing the supporting material can become difficult and even damage the structural integrity of the stent. The printing nozzle that moves within a small range can produce drawing and bonding effects of the TPU material, resulting in reduced printing accuracy. The axial lengths of the four types of stents are shown in Table 2.

### 2.2. Mechanical Property Simulation of the Vascular Stent

After the stent is implanted into a blood vessel, it will follow the deformation as the blood vessel bends. Usually, we use flexibility to measure the performance of stents following deformation. Stents with excellent flexibility can better coordinate the deformation between stents and arterial blood vessels and relieve the inflammatory reaction caused by the interaction between the stent’s outer surface and the blood vessel’s inner wall effectively. We can evaluate the compliance of the vascular stent structure with the law between equivalent stress and bending radian of the stent structure. In addition, an exemplary stent should also have excellent radial support performance to dilate blood vessels better. This study investigated the compliance and support performance of various stents using unilateral bending and radial compression simulation, respectively. At the same time, the influence of geometric parameters of the vascular stent element, such as edge width and wall thickness, on the different structures of the vascular stent was discussed.

#### 2.2.1. The Simulation Method of Unilateral Bending and Radial Compression

The unidirectional bending deformation of 7 mm and the radial compression deformation of 1 mm were selected because these values were within the normal deformation range of the aortic artery, iliac artery, and subclavian artery.

**Figure 2:**
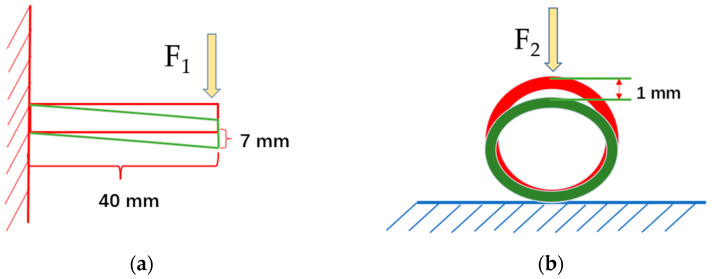
(**a**) The setting of the vascular stent bending model; (**b**) the setting of the vascular stent radial compression model.

To simulate the axial compliance of stents, the first step is to import the actual material properties into the engineering data module and assign them. The mechanical properties of the material settings are shown in Table 3. Subsequently, the four types of stents are meshed, with particular attention paid to mesh refinement in areas of complex geometries to ensure accuracy. In order to simulate uniform stress distribution during loading, multi-point constraints (MPCs) are created, equating the end faces of the stents to two points along the axial direction. The distance between these points represents the length of the stent. In the static structural analysis module, a fixed boundary condition is applied to constrain the movement at the left equivalent point of the stent. At the same time, a displacement command is used to impose a vertical displacement of 7 mm at the right equivalent point, simulating the bending deformation of the stent around its axis [52]. A concentration force, *F*_1_, is applied on the right end, as shown in Figure 2a. This approach enables us to solve the maximum equivalent stress in stents with varying edge widths and wall thicknesses under bending conditions.

In order to evaluate the radial support performance of the stent, the material assignment and meshing of the stent are carried out first. A column coordinate system is defined with the outer wall of the stent as the datum plane, and the tangential degree of freedom of each stent unit inside the stent is constrained. The axial degree of freedom of the stent unit inside the stent is constrained simultaneously to ensure that the stent can only be freely deformed in the radial direction. The rigid body displacement of the stent in other directions is avoided. After the constraint is applied in the cylindrical coordinate system, a displacement of 1 mm pointing to the axis is applied to the outer wall to cause the stent to shrink radially. A vertical downward force, F2, acts on the highest point of the vascular stent, as shown in Figure 2b. The maximum equivalent stress of the stent with different edge widths and wall thicknesses is solved.

We can evaluate the advantages and disadvantages of the thin-walled porous tubular structure of the vascular stent by structural stiffness. On the one hand, the compliance of the stent structure can be measured from the relationship between the equivalent stress and bending radian. On the other hand, the support performance of the stent structure can be evaluated from the relationship between the equivalent stress and radial compression. Under the same deformation condition, higher equivalent stress reflects more vigorous bending and compressive stiffness. This stress and strain connection shows the flexibility and support performance of the stents.

#### 2.2.2. The Simulation of Unilateral Bending and Radial Compression of Various Stents with Different Edge Width and Wall Thickness

**Figure 3:**
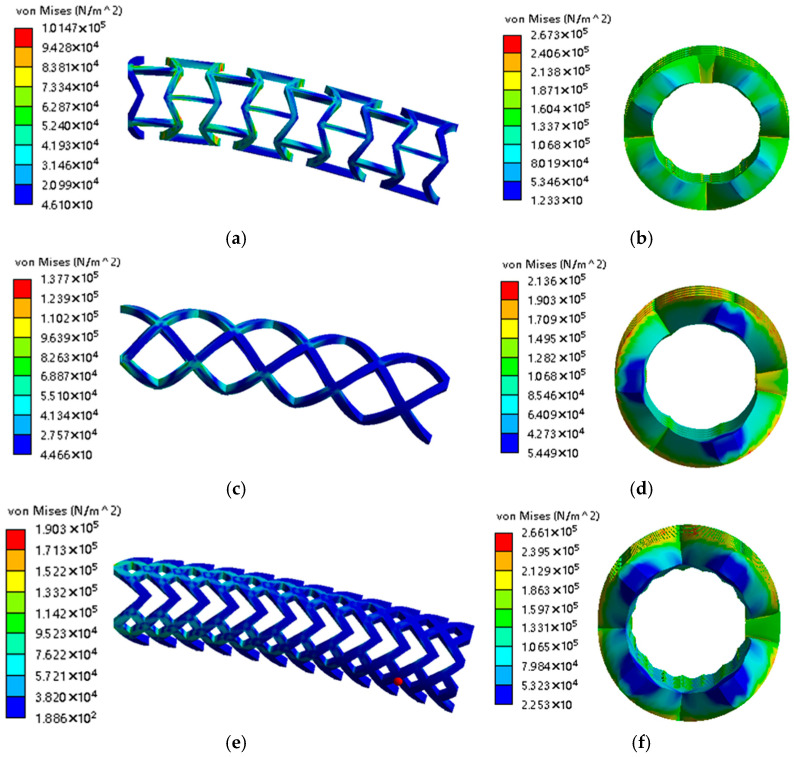

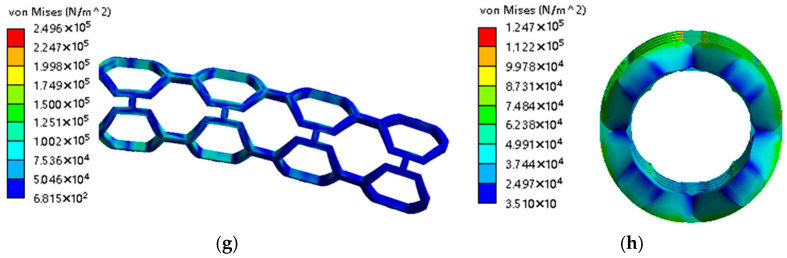
Simulation results: (**a**) unilateral bending at inner hexagonal stent; (**b**) radial compression at inner hexagonal stent; (**c**) unilateral bending at quadrilateral stent; (**d**) radial compression at quadrilateral stent; (**e**) unilateral bending at arrow stent; (**f**) radial compression at arrow stent; (**g**) unilateral bending at hexagonal stent; (**h**) radial compression at hexagonal stent.

Different stents often reflect different mechanical properties under different edge widths and wall thicknesses. The initial edge width of the four types of stents was set at 1.0 mm. Four types of stents with an edge width of 1.0 mm to 1.6 mm were designed with a gradient of 0.2 mm. From the finite element simulation, the maximum equivalent stress under radial compression and unilateral bending conditions is obtained as follows. At the same time, the initial wall thickness of the stent was set at 1.0 mm, and four types of stents with a wall thickness from 1.0 mm to 1.3 mm were designed with a gradient of 0.1 mm. The simulation calculation also obtains the maximum equivalent stress under the conditions of radial compression and unilateral bending. Figure 3 is a demonstration of the simulation result.

(1) A simulation of four kinds of unilateral bent stents under the conditions of an edge width of 1.0 mm and wall thickness of 1.2 mm is produced.
(2) The top-side compression simulation of four kinds of stents under the conditions of an edge width of 1.0 mm and wall thickness of 1.2 mm is shown in Figure 3. From top to bottom, there is an inner hexagonal stent, quadrilateral stent, arrow stent and hexagonal stent.

The simulation results of the unilateral bending and radial compression of the four types of stents with a wall thickness of 1.2 mm and different edge widths are shown in Table 4 and Table 5.

**Figure 4:**
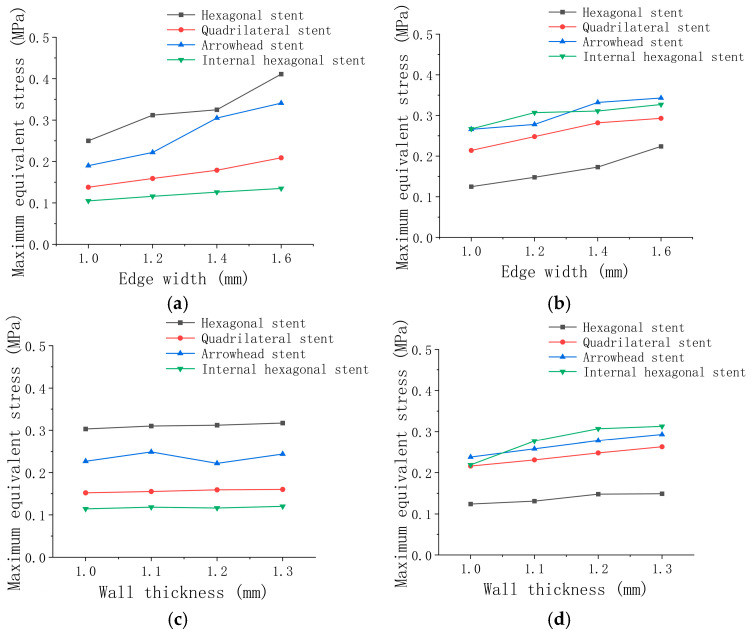
Simulation results of maximum equivalent stress for the edge widths and wall thicknesses: (**a**) unilateral bending at different edge widths; (**b**) radial compression at different edge widths; (**c**) unilateral bending at different wall thicknesses; (**d**) radial compression at different wall thicknesses.

Discussion and analysis: In the unilateral bending simulation, the stent unit on the upper surface of the stent is subjected to horizontal tensile force, and the stent unit on the lower surface of the stent is subjected to horizontal compressive force. All the vascular stent units were subjected to vertical compressive force in radial compression. From the unilateral bending simulation in Figure 4a, the maximum stress of the four types of vascular stents shows an upward trend with the increase in edge width. The maximum stress of the hexagonal and quadrilateral stents shows a stable linear change. Compared with the arrow-shaped and hexagonal stent, the maximum equivalent stress of the inner hexagonal stent and the quadrilateral stent is the smallest under the same deformation conditions. As for the inner hexagonal element, the quadrilateral element is more prone to deformation under transverse tension and compression, which leads to a lower bending stiffness and good axial compliance of the stent. The stents exhibit a lower bending stiffness and excellent axial compliance. From Figure 4b, we can find that the radial compressive performance of the stent is significantly improved with the increase in edge width. Under the same edge width condition, the value of the equivalent stress of the internal hexagonal vascular stent is larger, showing stronger compressive stiffness and better support performance. In terms of stent types, the hexagonal stent with negative Poisson’s ratio structure shows excellent characteristics of high support and high flexibility compared with the other three types of stents, which shows the advantage of negative Poisson’s ratio structure. Based on the simulation results of the two stress conditions, we can see that increasing the edge width will improve the support’s compressive stiffness and bending stiffness. As a result, the flexibility of the stent is improved, while the support performance of the stent is sacrificed. The simulation results of the unilateral bending and radial compression of the four types of stents with edge widths of 1.2 mm and different wall thicknesses are shown in Table 6 and Table 7.

Discussion and analysis: Figure 4c shows that in the unilateral bending simulation, the internal hexagonal and quadrilateral elements are more prone to deformation under the same deformation condition. The equivalent stress generated by the internal hexagonal vascular stent and the quadrilateral stent is smaller, showing lower bending stiffness. Both types of stents show excellent flexibility. It can be seen from Figure 4d that in the radial compression simulation, four kinds of stent units are subjected to vertical pressure under the same deformation conditions. The inner hexagonal and arrow-shaped units are more difficult to compress. The two kinds of vascular stents exhibited the largest equivalent stress, showing a stronger compressive stiffness and a better supporting performance. From the point of view of the wall thickness, increasing the wall thickness has no obvious influence on the bending performance of the stent, and it will not improve the stent’s bending stiffness and weaken the stent’s compliance performance.

### 2.3. Fabrication of the Artificial Vascular Stent

TPUs from Polymaker are selected as the printing material for fabricating the vascular stent using FFF technology. The corresponding mechanical properties of the commercial materials are shown in Table 3.

**Figure 5:**
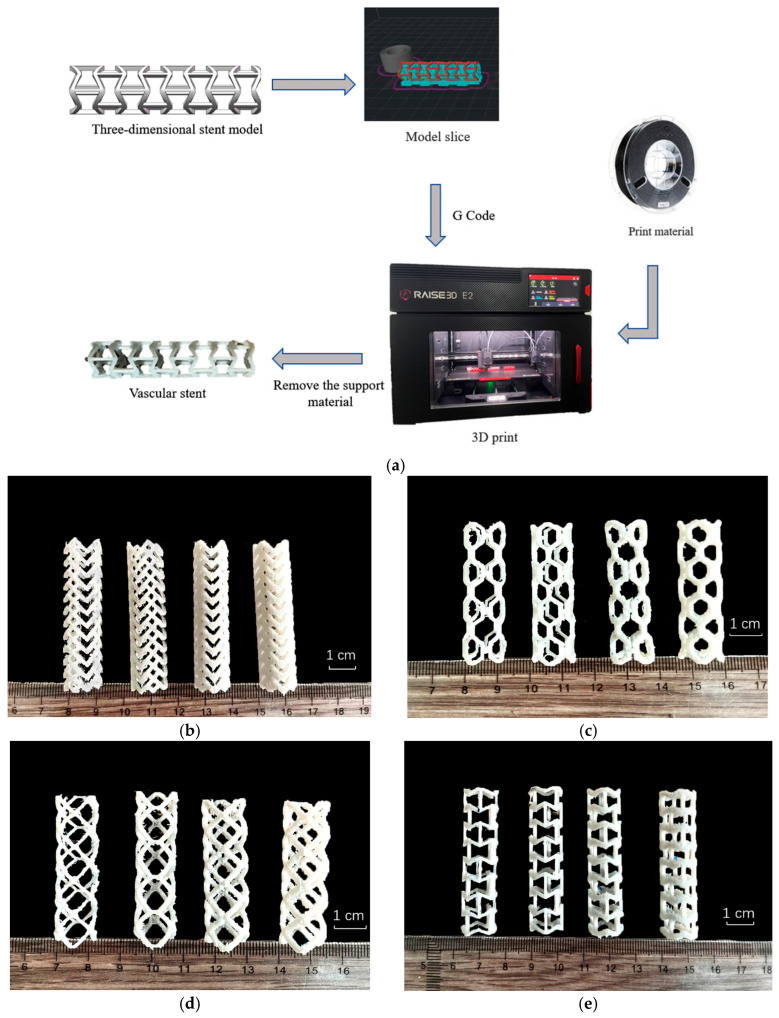

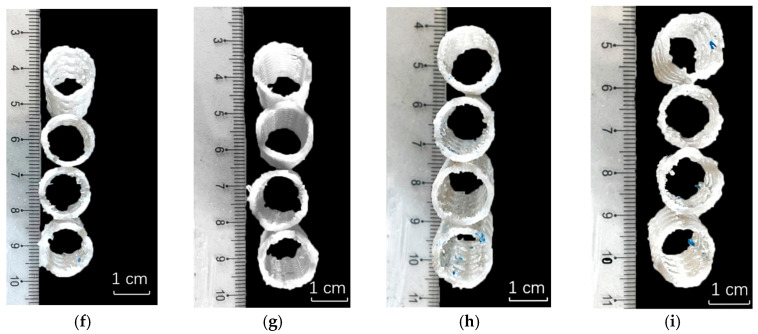
The production process and physical objects of stents. (**a**) Preparation process of TPU vascular stent by fused filament fabrication (**b**) Arrow-shaped stents with a wall thickness of 1.2 mm and edge widths of 1.0 mm, 1.2 mm, 1.4 mm, and 1.6 mm from left to right. (**c**) Hexagonal stents with a wall thickness of 1.2 mm and edge widths of 1.0 mm, 1.2 mm, 1.4 mm, and 1.6 mm from left to right. (**d**) Quadrilateral stents with a wall thickness of 1.2 mm and edge widths of 1.0 mm, 1.2 mm, 1.4 mm, and 1.6 mm from left to right. (**e**) Internal hexagonal stents with a wall thickness of 1.2 mm and edge widths of 1.0 mm, 1.2 mm, 1.4 mm, and 1.6 mm from left to right. (**f**) Hexagonal vascular stents with an edge width of 1.2 mm and a wall thickness of 1.0 mm, 1.1 mm, 1.2 mm, and 1.3 mm from left to right. (**g**) Arrow-shaped vascular stents with an edge width of 1.2 mm and a wall thickness of 1.0 mm, 1.1 mm, 1.2 mm, and 1.3 mm from left to right. (**h**) Internal hexagonal vascular stents with edge widths of 1.2 mm and wall thicknesses of 1.0 mm, 1.1 mm, 1.2 mm, and 1.3 mm from left to right. (**i**) Quadrilateral vascular stents with an edge width of 1.2 mm and a wall thickness of 1.0 mm, 1.1 mm, 1.2 mm, and 1.3 mm from left to right.

The Raise3D E2 high-precision printer was produced by Shanghai Fuzhi Company. The printing process is FFF, the nozzle size of the printing machine is (0.40 ± 0.02) mm, and the fused filament direction is 90 degrees vertical. The basic printing parameters are set as shown in Table 8. We have tested the mechanical properties of TPU, and the results are within the range provided by the supplier. Simulation studies are carried out based on the test results. The forming process of the vascular stent is shown in Figure 5a.

The three-dimensional digital model was first imported into the software IdeaMaker 4.3.3.6560 for the slicing. At the same time, the coiled filament TPU material was assembled into the printer. The melt deposition command was executed to form the structure according to the layer thickness of 0.1 mm. The supporting material was physically removed after the fabrication. The forming effect of vascular stents with different structures and geometric dimensions is shown in Figure 5.

## 3. The Mechanical Performance Evaluation of the Vascular Stents

### 3.1. The Unilateral Bending and Radial Compression Performance

To compare with the simulation results, all stents are printed and molded under the same printing parameters. The volume of the stents was enlarged eight times, considering the forming accuracy of the printer. The actual mechanical properties of the printed vascular stents are measured. The test items are the unilateral bending experiment and radial compression experiment. The experimental instrument is a universal tensile machine produced by Chatillon Company in the United States. In order to test the axial compliance of the stent, one side of the stent was fixed. An external concentrated force perpendicular to the stent was applied at a distance of 40 mm from the fixed end of the stent, which produced a vertical displacement of 7 mm. In order to test the radial support performance of the stent, the stent was placed flat on the experimental platform. A concentrated force was applied to the upper part of the stent to make one side of the stent move vertically by 1 mm downward. During the test, the required forces of four kinds of vascular stents during bending and compression deformation were obtained through the digital display screen on the tensile machine. To ensure the repeatability of the experiment results, we 3D printed three samples of each structure, measured the mechanical properties of each sample three times, and took the average values as the results. The experimental data were collected as shown in Table 9, Table 10, Table 11 and Table 12.

Discussion and analysis: Under the conditions of the same wall thickness and different edge widths, the inner hexagonal stent and the quadrangular vascular stent required a minor deformation force and showed better flexibility. The arrow stent needed the most deformation force and showed the worst flexibility. In the radial compression experiment, the arrow-shaped vascular stent and the inner hexagonal stent showed the best compression performance. In contrast, arrow-shaped stents showed high support performance and low flexibility. The inner hexagonal stent performed well in both tests, with high flexibility and excellent support performance. For three types of stents, the inner hexagonal stent, quadrilateral stent, and hexagonal stent, increasing the edge width could effectively strengthen the stents’ support performance without affecting the stents’ compliance performance. The edge width influences the arrow-shaped vascular stent more because of its dense cell arrangement, which still gives high support performance and low compliance characteristics.

**Figure 6:**
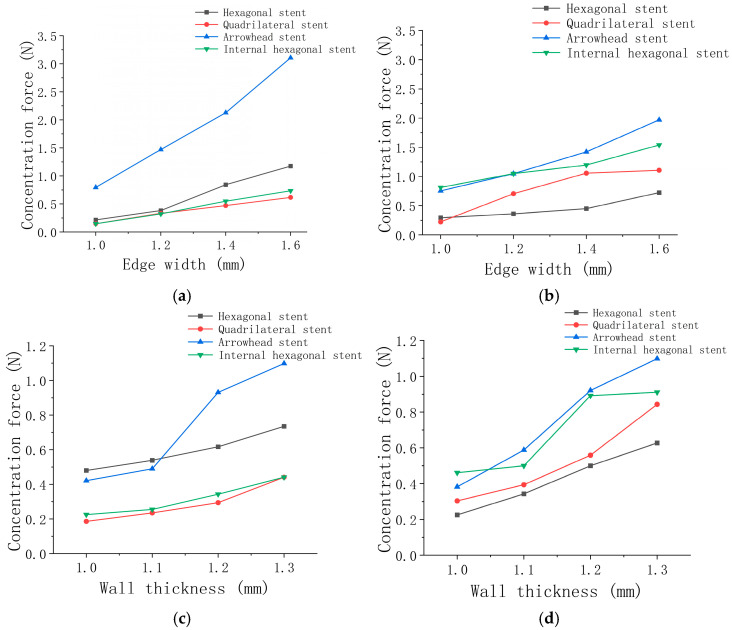
Experimental results: (**a**) unilateral bending at different edge widths; (**b**) radial compression at different edge widths; (**c**) unilateral bending at different wall thicknesses; (**d**) radial compression at different wall thicknesses.

Discussion and analysis: From the changing trend reflected in Figure 6c,d, we find that increasing the wall thickness can improve the radial compression resistance of the stent more than increasing the edge width. Based on the same edge width and wall thickness, the force required for the axial deformation of quadrangular support and inner hexagonal support is the smallest, showing good compliance. The hexagonal vascular stent shows low compliance and support compared with the previous simulation results. In the radial compression experiment, the arrow-shaped vascular stent and the inner hexagonal stent show the best supporting performance. It is worth mentioning that the arrow-shaped stent shows both muscular axial stiffness and muscular radial compression stiffness due to the low porosity of its outer surface, which once again verifies its high supporting performance and low compliance. However, the inner hexagonal stent with negative Poisson’s ratio structure shows high flexibility and a similar support performance to the arrow-shaped vascular stent.

### 3.2. Support Force Measurement of the Vascular Stent

**Figure 7:**
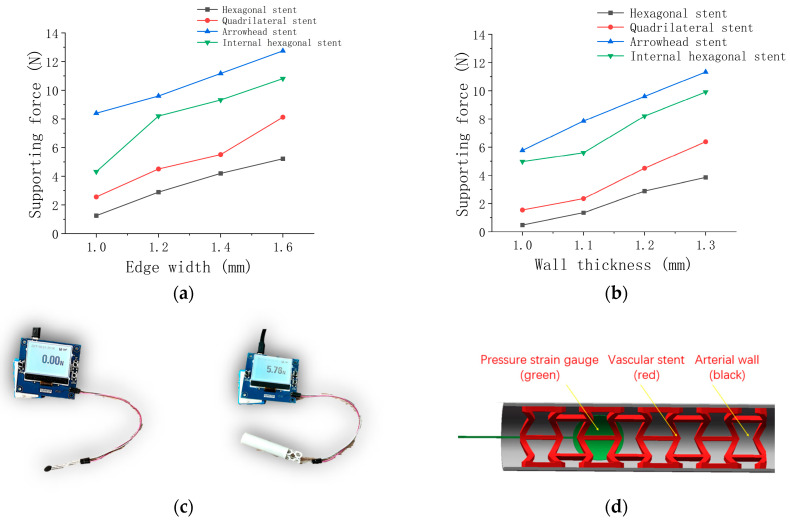
Test results and method: (**a**) stents with different edge widths; (**b**) stents with different wall thicknesses; (**c**) membrane pressure sensor and vascular stent; (**d**) testing the support force of vascular stent.

As shown in Figure 7c, the film pressure sensor was used to verify the supporting force generated after stent implantation [55]. The film pressure sensor detected the actual supporting force generated by each stent with the same wall thickness and the edge width was detected through the film pressure sensor.

The experimental design: The simulated blood vessel was printed with TPU by a 3D printing machine, considering that the elastic modulus of TPU material is 6.17 MPa and the elastic modulus of the blood vessel is 5 MPa. After the stent was placed in the simulated blood vessel, the thin-film strain gauge with a thickness of 0.1 mm was placed at the contact plane between the outer surface of the stent and the inner wall of the simulated blood vessel. The thin-film pressure sensor will display the bearing stress of the strain gauge, which is the supporting force provided by each stent. The inner wall of the simulated blood vessel is 12 mm, and the outer diameter of all stents is 12.8 mm. The stents are implanted into the simulated blood vessel in a compressed state. The supporting force data of each stent are collected after implantation. A demonstration of the experiment is shown in Figure 7d.

Discussion and Analysis: From the test data in Figure 7a,b, we know that increasing the wall thickness can improve the stent’s supporting force and the stent’s compression resistance more than increasing the edge width. The arrow-shaped support shows the highest compressive stiffness and the best supporting performance because of its low porosity and incompressibility. Quadrilateral stents and hexagonal stents perform poorly in the test of supporting force because of the poor resilience of stent structural units. The inner hexagonal stent with negative Poisson’s ratio structure shows a slightly worse supporting force performance than the arrow stent, showing the superiority of negative Poisson’s ratio unit structure. The measurement results of the independent variation of radial support force with edge width and wall thickness are summarized in Table 13 and Table 14.

### 3.3. Comparison of Simulation and Experimental Results

The geometric dimensions of the simulation experiments are different, resulting in the simulation results and the experimental results data not being in the same order of magnitude. We use vector normalization to process the data to make it in the same magnitude and compare the change trends. The formula used is as follows [56]:

(1) $$\;\;\;\;y_{i}=\frac{x_{i}}{\sum_{i=1}^{n}x_{i}},$$

where $y_{i}$ is the mapped value of the support reaction force; $x_{i}$ is the reaction force; and $\sum_{i=1}^{n}x_{i}$ is the sum of the reaction forces. The normalized mapping value of the support reaction force is defined as *F*.

**Figure 8:**
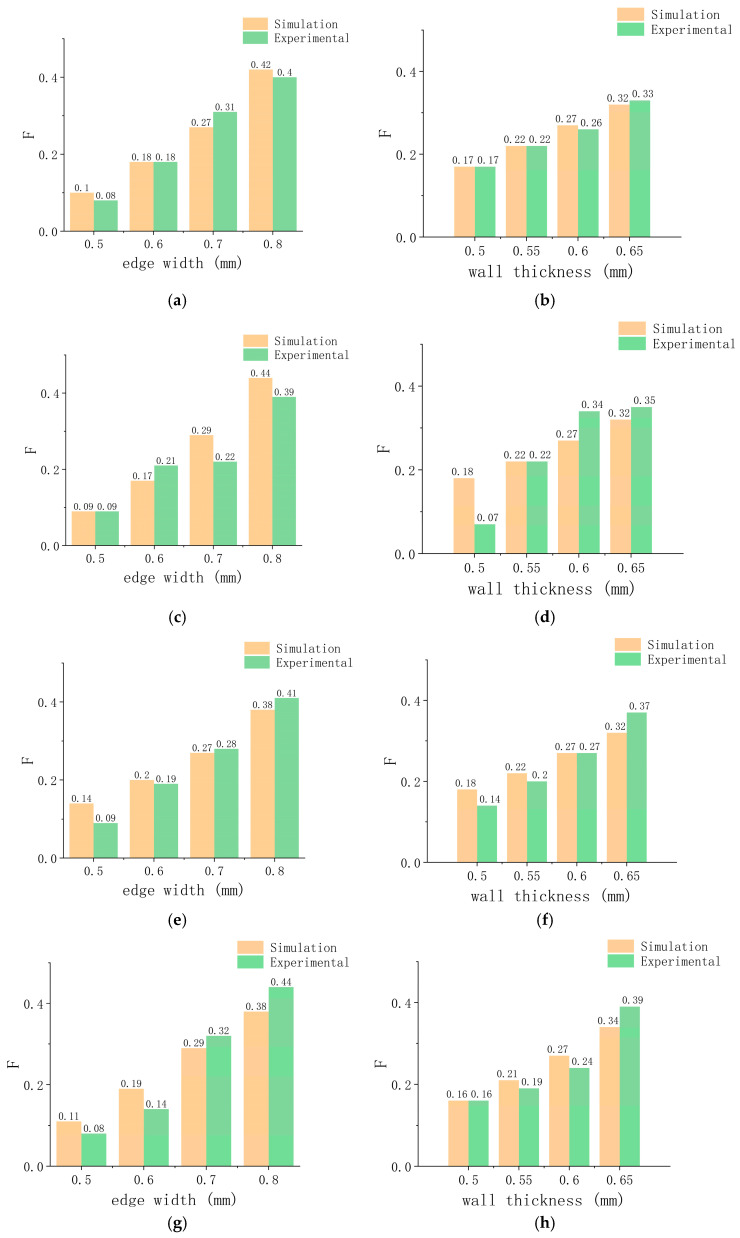
Comparison of unilateral bending simulation experiments: (**a**) internal hexagonal stent at different edge widths; (**b**) internal hexagonal stent at different wall thicknesses; (**c**) quadrilateral stent at different edge widths; (**d**) quadrilateral stent at different wall thicknesses; (**e**) arrowhead stent at different edge widths; (**f**) arrowhead stent at different wall thicknesses; (**g**) hexagonal stent at different edge widths; (**h**) hexagonal stent at different wall thicknesses.

**Figure 9:**
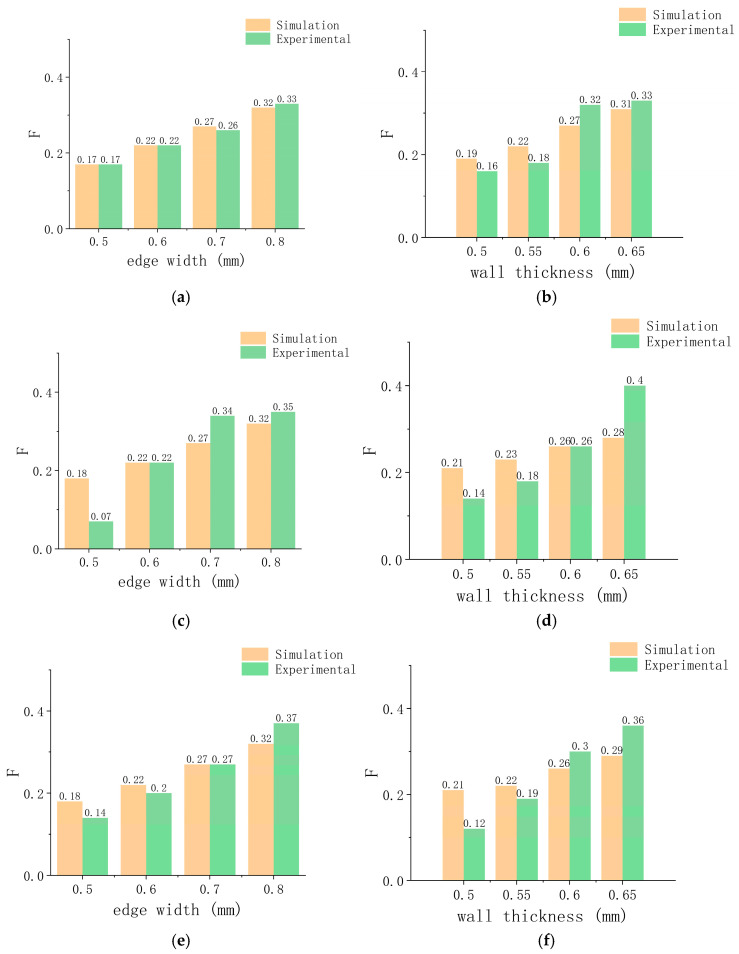

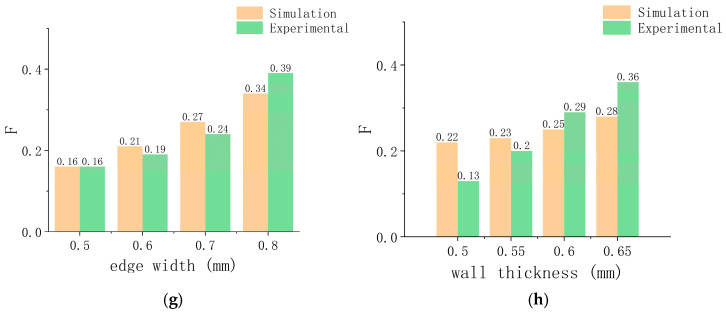
Comparison of radial compression simulation experiments: (**a**) internal hexagonal stent at different edge widths; (**b**) internal hexagonal stent at different wall thicknesses; (**c**) quadrilateral stent at different edge widths; (**d**) quadrilateral stent at different wall thicknesses; (**e**) arrowhead stent at different edge widths; (**f**) arrowhead stent at different wall thicknesses; (**g**) hexagonal stent at different edge widths; (**h**) hexagonal stent at different wall thicknesses.

Figure 8 shows the simulation and experimental comparison of the normalized mechanical properties of the axial bending of the vascular stents. Figure 9 shows the simulation and experimental comparison of the radial support performances of vascular stents after normalization. The difference between the calculation results and the measurement results might be that the mechanical properties of TPU material are set to be uniform in the simulation calculation. Due to the layer-by-layer deposition 3D printing process, the actual fabricated vascular stents have laminate microstructures, which lead to anisotropy in their mechanical properties. However, whether it is the radial support ability or axial compliance of vascular stents, their trends with a variation in edge length and wall thickness are consistent with the simulation and experimental results.

### 3.4. Comparison of Equivalent Stress Among TPU Stents, Commercial Stents, and Native Vessels

**Figure 10:**
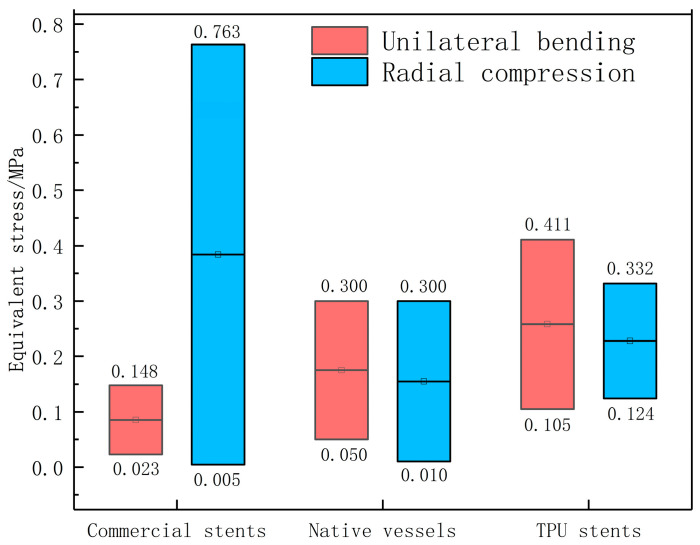
The comparison of equivalent stress among TPU stents, commercial stents, and native vessels.

The typical commercial vascular stents’ unilateral bending equivalent stress range is 2.3 × 10^−2^ to 1.48 × 10^−1^ MPa, and the radial compression equivalent stress range is 4.56 × 10^−3^ to 7.63 × 10^−1^ MPa [57]. The equivalent stress range for bending in human blood vessels is 5.2 × 10^−2^ to 3.11 × 10^−1^ MPa, and the equivalent stress range for support is 1.3 × 10^−2^ to 3.14 × 10^−1^ MPa. The TPU stents’ unilateral bending equivalent stress range of vascular stents is 1.05 × 10^−1^ to 4.11 × 10^−1^ MPa, and the radial compression equivalent stress range is 1.24 × 10^−1^ to 3.32 × 10^−1^ MPa, as shown in Figure 10. Compared to commercial vascular stents, TPU stents have a wider range of compatibility with human blood vessels in terms of axial bending mechanical properties. To achieve better deformation coordination and consistency between TPU stents and human blood vessels, the geometry parameters of the TPU stents are recommended to be as follows: the implantable range of the inner hexagon is 1.0 to 1.2 mm in edge width and 1.0 to 1.2 mm in wall thickness; the implantable range of the quadrilateral is 1.0 to 1.6 mm in edge width and 1.0 to 1.3 mm in wall thickness; the implantable range of the arrow-shaped structure scaffold is 1.0 to 1.2 mm in edge width and 1.0 to 1.2 mm in wall thickness; and the implantable range of the hexagonal stent scaffold is 1.0 mm in edge width and 1.0 to 1.2 mm in wall thickness.

### 3.5. Failure Analysis of the Vascular Stent

After printing out four types of vascular stents, we tested their unilateral bending with large deflection. We applied an increasing displacement deformation on one side of the stent and finally found that the edge joint near the fixed end of the stent failed due to its high structural stiffness, which the stress concentration may cause.

#### 3.5.1. Failure of Vascular Stent from the Structural Point of View

**Figure 11:**
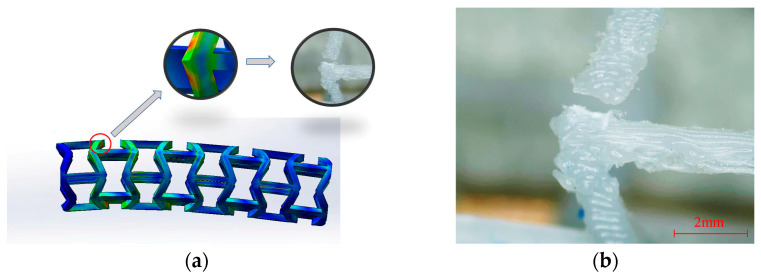
(**a**) Stress concentration of the stent leads to a fracture; (**b**) fracture failure diagram of TPU vascular stent sample.

Taking the internal hexagonal vascular stent as an example, the failure of the stent often occurs at the position with the maximum stress, which is at the border junction of the stent. Because of the high relative stiffness of the stent at the border junction, its deformation ability is weak, and it is more prone to fracture than other parts, as shown in Figure 11a,b.

#### 3.5.2. The Failure Mechanism from the Perspective of Deposition Mode

FFF, also known as fuse deposition, is used to heat and melt filamentary hot-melt materials, extrude them through a fine nozzle, and stack printed materials layer by layer to finally form unistructural materials. When printing materials are stacked layer by layer, the adhesion between layers depends on the materials’ melting. The mechanical properties are lower than the complete materials and show anisotropic characteristics, as shown in Figure 11b. When the internal shear stress is perpendicular to the deposition direction, the joint between layers is prone to fracture failure.

## 4. Conclusions

Vascular stents with different structural units have different mechanical properties. Under the same edge width and wall thickness, the hexagonal stent with negative Poisson’s ratio structure has better comprehensive performance than the arrow stent and quadrilateral stent. The inner hexagonal stent can maintain high flexibility and excellent support performance. In contrast, the arrow stent shows the best support performance and poor flexibility, probably due to the problematic compression characteristics of its dense cell arrangement and arrow stent. The quadrilateral stent shows excellent flexibility and poor radial support performance. Hexagonal stents show low flexibility and low support performance.

From the point of view of edge width and wall thickness, increasing the edge width has a greater impact on stents with a denser arrangement of stent units. This will greatly improve the support performance of stents but also reduce their flexibility. Increasing the wall thickness will improve the radial support performance of the stent, but it will only slightly reduce the compliance performance of the stent.

With an increase in edge width, the bending stiffness of the inner hexagonal stent and the quadrilateral stent is smaller than that of the arrow stent and the hexagonal stent under the same deformation conditions, showing good axial flexibility, and the axial compliance of the arrow stent decreases significantly with an increase in edge width. With an increase in wall thickness, the bending stiffness of quadrilateral stents, hexagonal stents and inner hexagonal stents did not increase significantly, and their flexibility did not decrease significantly. In contrast, the arrow stent showed the worst suppleness. With the increase in edge width, the arrow stent and the inner hexagonal stent have the best compressive performance, the compressive performance of the quadrilateral stent increases steadily, and the hexagonal stent reflects the worst supporting performance. With the increase in wall thickness, the compressive performance of the inner hexagonal stent and the arrow stent is continuously enhanced, and the supporting force of the quadrilateral stent increases linearly, and its compressive performance is weaker than that of the arrow stent and the inner hexagonal stent and stronger than that of the hexagonal stent. On the whole, increasing the edge width and wall thickness will improve the compressive performance of the stent, and the increase in edge width will be greater.

In the supporting force test, the maximum supporting force of the arrow-shaped stent is 12.75 N, and the maximum supporting force of the inner hexagonal vascular stent is 10.81 N. In a follow-up study, the supporting force of the stent can be further improved by selecting a stronger TPU material and polymer metal composites. The structure of the stent unit is also preferred for optimization.

The stent’s fracture point typically occurs at the edge of the vascular stent and the edge joint because the edge joint is prone to stress concentration due to its strong structural stiffness. From the perspective of fracture failure mode, the fracture’s cross-section tends to occur perpendicular to the 3D printing deposition direction due to the anisotropy of mechanical properties of the samples processed by FFF technology.

The simulation results of the four types of stents are slightly different from the actual test results, but the mechanical properties of each stent tend to be consistent in the test. This difference might be because the stent defined in the simulation software is a complete entity. In contrast, the vascular stent used in the experiment is stacked by melting deposition. The stent fabricated by this printing process tends to give an anisotropic performance of the mechanical properties.

This article designed and 3D printed vascular stents based on four units’ structures: inner hexagon, arrow, quadrilateral, and outer hexagon. The relationship between the geometric parameters of the unit structure and the macroscopic mechanical properties in vascular stents was verified through simulation and experimental measurements. The research on the deformation coordination between vascular stents and vascular tissues provides a theoretical design basis for customized clinical diagnosis and treatment plans. In practical clinical applications, many factors still need to be considered. For example, the surface modification of vascular stents, the precision of forming vascular stents with smaller inner diameters, and the blood compatibility of vascular stent materials are still worth further exploration.

## Figures and Tables

**Table 1:** Geometric dimension parameters of the unit structures.

$\theta_{1}$	$\theta_{2},\theta_{3}$	$H_{1}$	D	$L_{1}$	$W_{1}$	$L_{2},W_{2},\emptyset_{d}$	$L_{3}$	$L_{4}$
70°	90°	8 mm	6 mm	3 mm	1.5 mm	4 mm	3.2 mm	2.7 mm

**Table 2:** Length of four types of vascular stents.

Types of Stents	Internal Hexagonal Stent	Arrow Stent	Quadrilateral Stent	Hexagonal Stent
Length of stent (mm)	50.4	50.8	51.7	47.8

**Table 3:** Mechanical properties of TPU structures.

Property	Test Method	Typical Value
100% modulus (X-Y)	ISO37 [53], GB/T528 [54]	6.17 ±0.19 MPa
Tensile strength (X-Y)	ISO37, GB/T528	30.0±0.66 MPa
Elongation at break (X-Y)	ISO37, GB/T528	586.8±15.3%

**Table 4:** Simulation results of unilateral bending with different edge widths when wall thickness is 1.2 mm.

	Edge Width (mm)	1.0	1.2	1.4	1.6
Maximum Equivalent Stress (MPa)					
Internal hexagonal stent		0.105	0.116	0.126	0.135
Quadrilateral stent		0.138	0.159	0.179	0.209
Arrowhead stent		0.190	0.222	0.305	0.341
Hexagonal stent		0.250	0.312	0.325	0.411

**Table 5:** Simulation results of radial compression with different edge widths when wall thickness is 1.2 mm.

	Edge Width (mm)	1.0	1.2	1.4	1.6
Maximum Equivalent Stress (MPa)					
Internal hexagonal stent		0.267	0.307	0.311	0.327
Quadrilateral stent		0.214	0.248	0.282	0.293
Arrowhead stent		0.266	0.278	0.332	0.343
Hexagonal stent		0.125	0.148	0.173	0.224

**Table 6:** Simulation results of unilateral bending with different wall thicknesses when edge width is 1.2 mm.

	Wall Thickness (mm)	1.0	1.1	1.2	1.3
Maximum Equivalent Stress (MPa)					
Hexagonal stent		0.303	0.310	0.312	0.317
Quadrilateral stent		0.152	0.155	0.159	0.160
Arrowhead stent		0.227	0.249	0.222	0.244
Internal hexagonal stent		0.114	0.118	0.116	0.120

**Table 7:** Simulation results of radial compression with different wall thicknesses when edge width is 1.2 mm.

	Wall Thickness (mm)	1.0	1.1	1.2	1.3
Maximum Equivalent Stress (MPa)					
Hexagonal stent		0.124	0.131	0.148	0.149
Quadrilateral stent		0.216	0.231	0.248	0.263
Arrowhead stent		0.238	0.258	0.278	0.293
Internal hexagonal stent		0.219	0.277	0.307	0.313

**Table 8:** Basic printing parameters.

Print Layer Thickness (mm)	Print Speed (mm/s)	Printing Temperature (°C)	Filling Percentage (%)	Build Platform Temperature (°C)
0.1	60	220	100	60

**Table 9:** Experimental results of unilateral bending with different edge widths when wall thickness is 1.2 mm.

	Edge Width (mm)	1.0	1.2	1.4	1.6
Concentration Force (N)					
Hexagonal stent		0.216	0.382	0.843	1.176
Quadrilateral stent		0.148	0.333	0.470	0.617
Arrowhead stent		0.794	1.470	2.127	3.107
Internal hexagonal stent		0.147	0.323	0.549	0.735

**Table 10:** Experimental results of radial compression with different edge widths when wall thickness is 1.2 mm.

	Edge Width (mm)	1.0	1.2	1.4	1.6
Concentration Force (N)					
Hexagonal stent		0.294	0.361	0.451	0.725
Quadrilateral stent		0.225	0.706	1.058	1.107
Arrowhead stent		0.755	1.049	1.421	1.970
Internal hexagonal stent		0.813	1.050	1.196	1.539

**Table 11:** Experimental results of unilateral bending with different wall thicknesses when edge width is 1.2 mm.

	Wall Thickness (mm)	1.0	1.1	1.2	1.3
Concentration Force (N)					
Hexagonal stent		0.480	0.539	0.617	0.735
Quadrilateral stent		0.186	0.235	0.294	0.441
Arrowhead stent		0.421	0.490	0.931	1.098
Internal hexagonal stent		0.225	0.255	0.343	0.441

**Table 12:** Experimental results of radial compression with different wall thicknesses when edge width is 1.2 mm.

	Wall Thickness (mm)	1.0	1.1	1.2	1.3
Concentration Force (N)					
Hexagonal stent		0.225	0.343	0.500	0.627
Quadrilateral stent		0.304	0.394	0.559	0.843
Arrowhead stent		0.382	0.588	0.921	1.098
Internal hexagonal stent		0.461	0.500	0.892	0.911

**Table 13:** Test results of support force under different edge widths when wall thickness is 1.2 mm.

	Edge Width (mm)	1.0	1.2	1.4	1.6
Supporting Force (N)					
Hexagonal stent		1.25	2.89	4.20	5.22
Quadrilateral stent		2.56	4.51	5.51	8.12
Arrowhead stent		8.40	9.60	11.17	12.75
Internal hexagonal stent		4.32	8.20	9.32	10.81

**Table 14:** Test results of support force under different wall thicknesses when edge width is 1.2 mm.

	Wall Thickness (mm)	1.0	1.1	1.2	1.3
Supporting Force (N)					
Hexagonal stent		0.48	1.35	2.89	3.85
Quadrilateral stent		1.55	2.35	4.51	6.38
Arrowhead stent		5.77	7.85	9.60	11.33
Internal hexagonal stent		4.97	5.60	8.20	9.91

## Data Availability

The original contributions presented in the study are included in the article, further inquiries can be directed to the corresponding author.
